# PCaLiStDB: a lifestyle database for precision prevention of prostate cancer

**DOI:** 10.1093/database/baz154

**Published:** 2020-01-16

**Authors:** Yalan Chen, Xingyun Liu, Yijun Yu, Chunjiang Yu, Lan Yang, Yuxin Lin, Ting Xi, Ziyun Ye, Zhe Feng, Bairong Shen

**Affiliations:** 1 Center for Systems Biology, Soochow University, Suzhou 215006, China; 2 Department of Medical Informatics, School of Medicine, Nantong University, Nantong 226001, China; 3 School of Nanotechnology, Suzhou Industrial Park Institute of Services Outsourcing, Suzhou 215123, China; 4 Department of Urology, The First Affiliated Hospital of Soochow University, Suzhou 215006, China; 5 Institutes for Systems Genetics, West China Hospital, Sichuan University, No.17 Gaopeng Avenue, Chengdu 610041, China

## Abstract

The interaction between genes, lifestyles and environmental factors makes the genesis and progress of prostate cancer (PCa) very heterogeneous. Positive lifestyle is important to the prevention and controlling of PCa. To investigate the relationship between PCa and lifestyle at systems level, we established a PCa related lifestyle database (PCaLiStDB) and collected the PCa-related lifestyles including foods, nutrients, life habits and social and environmental factors as well as associated genes and physiological and biochemical indexes together with the disease phenotypes and drugs. Data format standardization was implemented for the future Lifestyle-Wide Association Studies of PCa (PCa_LWAS). Currently, 2290 single-factor lifestyles and 856 joint effects of two or more lifestyles were collected. Among these, 394 are protective factors, 556 are risk factors, 45 are no-influencing factors, 52 are factors with contradictory views and 1977 factors are lacking effective literatures support. PCaLiStDB is expected to facilitate the prevention and control of PCa, as well as the promotion of mechanistic study of lifestyles on PCa.

Database URL: http://www.sysbio.org.cn/pcalistdb/

## Introduction

The current belief is that most cancers are the result of inherited genetic abnormalities, yet 90% of malignancies are rooted in our lifestyle and environmental exposures. Lifestyle medicine is a paradigm for study of association between lifestyle and chronic disease through targeting at making realistic and progressive evidence-based lifestyle changes to reduce the risks of common modern diseases (mainly chronic, but potentially new lifestyle-related acute and infectious diseases) (1, 2). Research shows that more than 80% of chronic conditions could be avoided through the adoption of healthy lifestyle recommendations (3). Lifestyle precision medicine seeks rational prevention of the onset or progression of chronic disease, although the optimal interventions to achieve this have not been well documented in the literature (4).

Prostate cancer (PCa) is a heterogeneous disease with lethal and indolent phenotypes and is the most commonly diagnosed visceral cancer among men in most western countries (5, 6). Many epidemiological and case–control studies disclosed that there is a great link between lifestyles and PCa, such as body weight, smoking, dietary factors and also some other lifestyle-related diseases (hyperglycemia and dyslipidemia) (7–9). However, the evidence is still uncertain or inconclusive for many of the modifiable PCa-related lifestyle factors; systematic PCa lifestyle medicine research is even sparse.

Arab *et al*. (10) described that current smokers had a decreased risk of nonadvanced PCa (HR = 0.82, 95% CI: 0.77, 0.88), but an increased risk of fatal PCa (HR = 1.69, 95% CI: 1.25, 2.27). Brasky *et al*. (11) found that health products such as ginseng have no effect on the prevention of PCa. The association between folate status and risk of PCa is fuzzy and conflicting. Data from Arthur *et al*.’s study (12) regarding the potential influence of glucose on PSA levels may vary as one progress from the prediabetic phase to the diabetic phase. Lifestyle modifications like smoking cessation (13) and exercise (14) can decrease the risk of developing PCa.

However, lifestyles have very complex effects on the oncogenesis of PCa which could be protective or stimulative to the occurrence of PCa. These effects could be independent, joint or conditionally contradicted. Same lifestyles even play different roles in combination with different genotypes (15).

Based our knowledge, until now no specific database existed about disease-lifestyle association which can support lifestyle-wide association studies (LWAS) of disease. In particular, the benefit of lifestyle interventions in the prevention of disease progression has not been fully established. Most lifestyle studies are based on comprehensive nationwide databases (16). Exposome-Explorer (17) is the similar database but targeted application specific for biomarkers of exposure to dietary and environmental factors. With the paradigm shifting to prevention and participatory medicine (18), it is urgent to comprehensively investigate the impact of lifestyles on our health (19–21).

In order to promote the LWAS on PCa, we constructed the PCa-related lifestyle database (PCaLiStDB), aim to collect the PCa associated lifestyles and accelerate the lifestyle medicine for personalized PCa prevention and controlling.

## Materials and methods

### Data acquisition

#### Retrieval strategy.

A systematic review of the literature was performed by searching PubMed and Web of Science for PCa lifestyle studies published in English language, peer-reviewed journals before 1 February 2018. The search terms included ‘prostate cancer’, ‘risk factor’, lifestyle, vitamin, smoke, wine, tea, coffee, diet, dairy, nutrient^*^, alcohol, fruit, vegetable, environment, sleep, social, ‘sun exposure’, folate, ‘birth weight’, carotene, fiber, fried and carbohydrate. In addition, we reviewed studies in the reference lists of the retrieved studies to supply the baseline of this study and searched for other potentially eligible studies based on them.

#### Inclusion and exclusion criteria.

The inclusion criteria comprised the following: (i) the study was an in vivo or a human study; (ii) the literature was published after 2000; (iii) the study examined at least one item on etiology or diagnosis of PCa (all types of PCa); (iv) the study indicators had clear values; (v) the study with any effect size for which 95% confidence intervals (CIs) were provided or such information could be derived.

The exclusion criteria included the following: (i) the study used animal as subjects or in vitro study; (ii) the literature was published before 2000; (iii) the study combined PCa with other diseases; (iv) the study on the treatment, prognosis or recurrence of PCa without lifestyle effects; and (v) the study did not report any effect size with its 95% CIs or such information could not be derived of any influence factor.

#### Data screening and extraction.

After preliminary data filtering with reference to the inclusion and exclusion criteria, all included and excluded studies were tagged with specific annotations, such as different group names or symbols, to ensure data traceability and updates of later versions of PCaLiStDB. For example, data of some quantile studies without clear scope or boundaries were not included in the database this time; however, we retained and annotated them for the future updating.

We designed a pilot table which contains three sheets to extract the main information, baseline and outcome of each included study. Before data extraction, we developed a unified standardization for the data and annotation, so as to unify and speed up the process of data entry and make modification flexibly.

### Pre-processing and annotating

Due to the lack of definition and classification criteria for the overall lifestyles associated with a particular disease, we established a classification framework for PCa related lifestyles based on Cuzick’s research (22). According to the Third Expert Report of The World Cancer Research Fund/American Institute for Cancer Research (WCRF/AICR) (23, 24), we expanded the definition of lifestyles according to it.

**Figure 1:**
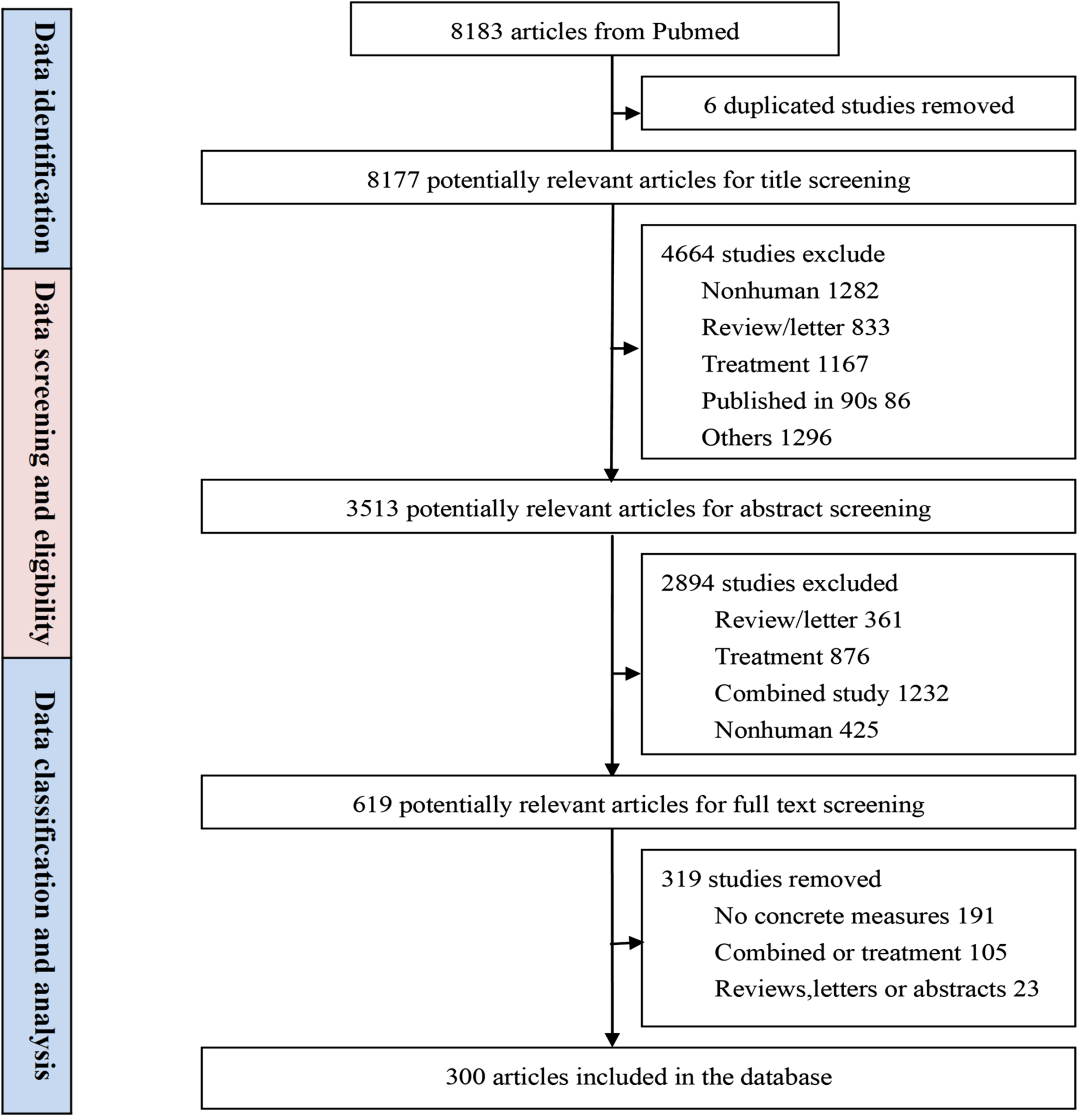
The schema of PCaLiStDB.

When completing the overall classification framework, we manually refined and classified all the lifestyles included in PCaLiStDB. We then qualitatively analyzed them to determine the attributes of the lifestyles based on statistical analysis and set the threshold as *P* value <0.05. So far, the effects of lifestyles in PCaLiStDB on PCa were classified as protective, risk, no influencing and contradictory. In addition, according to the *P* value and 95% CI, the levels of effects on PCa were scored as weak, moderate, strong and extremely strong correlation.

### Database implementation

The PCaLiStDB was constructed with MySQL server, Apache, PHP, HTML and JavaScript (25). The online database was implemented in the Windows operation system (64) (26). The quality of data was controlled and double-checked according to the standard of each stage to prevent data omission, duplication and ensuring data integrity.

## Results

### Entity and organization

A total of 8183 studies were identified from PubMed and Web of Science. After screening based on our inclusion and exclusion criteria, 300 articles were finally included in the PCaLiStDB. The working flow of the data collection for the PCaLiStDB is shown in Figure 1.

**Figure 2:**
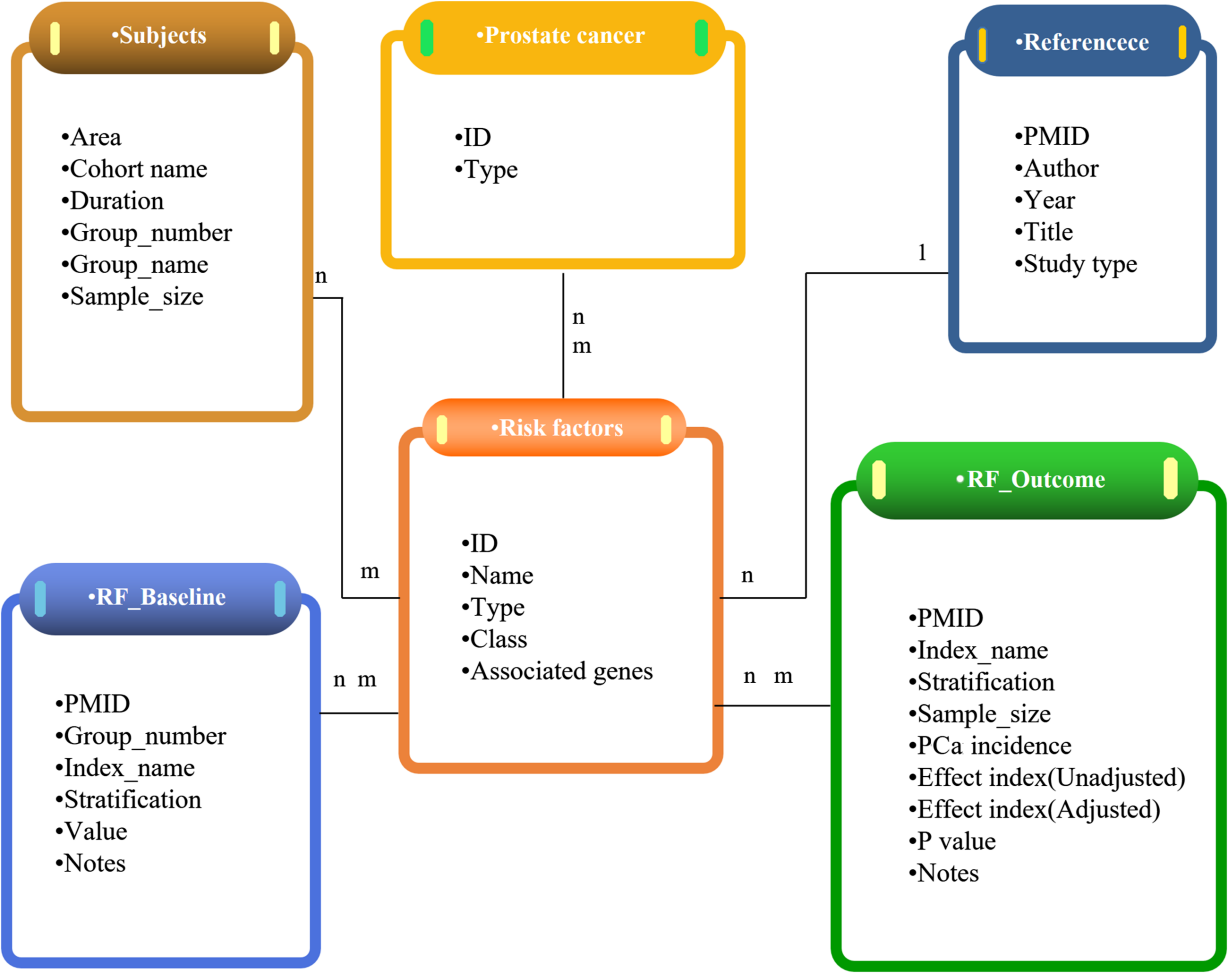
Overview of the structure of PCaLiStDB.

The information collected includes PMID, cohort name, study type, study duration, sample size and lifestyle types of each study. Moreover, the baseline data and outcomes related to the occurrence of PCa are all presented in the PCaLiStDB. Information in all the three sheets is correlated through the internal structure of PCaLiStDB which is shown in Figure 2.

### Interaction functions and demonstration

We consider that the users of this platform are not only medical staff but also the general public. While focusing on the science and sustainability of the PCaLiStDB, we pay attention to the simplicity of the interface and the convenience, the interactivity and the popularization of knowledge.

**Figure 3:**
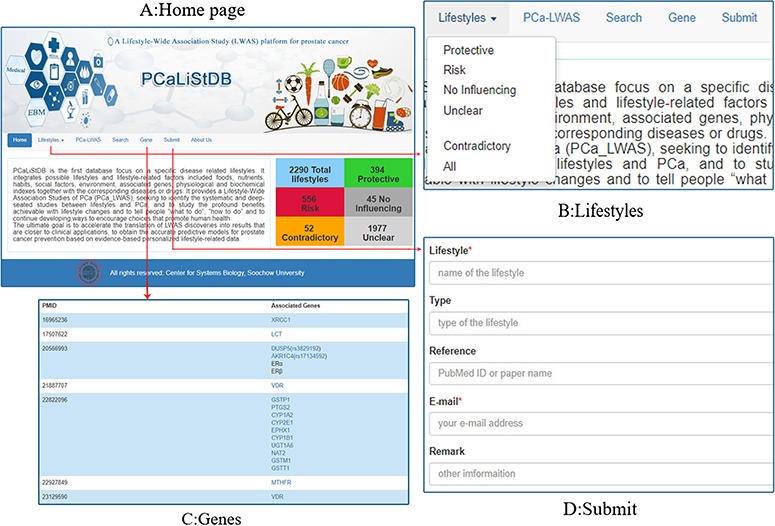
Functions and demonstration. **A**: Home page; **B**: lifestyles; **C**: genes; **D**: submit.

The ‘home’ page (Figure 3A) mainly introduces the contents and functions of the database platform, aiming to let consumers know our platform at a glance. The interface clearly lists the current number of PCa-related lifestyles and also shows the number of protective, risk, no influencing, contradictory and unclear type of lifestyles included in PCaLiStDB. At the same time, these attributes are annotated by different colors, which enable users to perceive the impact attributes intuitively.

The ‘Lifestyle’ (Figure 3B) has a drop-down menu including four manual qualitative analysis results of lifestyles in the database. ‘Protective’, ‘Risk’, ‘No influencing’, ‘Unclear’ menus list the corresponding lifestyles which is based on the *P* value. The ‘Contradictory’ refers to lifestyles that have been identified as different types of impact in different studies. Through these functional interfaces, users can quickly access specific information according to the type of lifestyle.

For the future genotype–phenotype–lifestyle analyses, we collected information for lifestyle associated genes. All the associated genes in PCaLiStDB are shown in the ‘Gene’ interface. In addition to the gene name and the corresponding PMIDs, all the genes have been standardized and automatically associated with the Gene and SNP database of NCBI. Through these databases, users can acquire the details of these genes (Figure 3C).

In order to achieve the sustainable development of the database, we collect relevant researches that may be omitted or added in the future through submit interface (Figure 3D). In order to reduce the workload and improve the enthusiasm to supplement, we only need them to provide the names of lifestyle and email, while lifestyle types, PMID and other information are optional.

**Figure 4:**
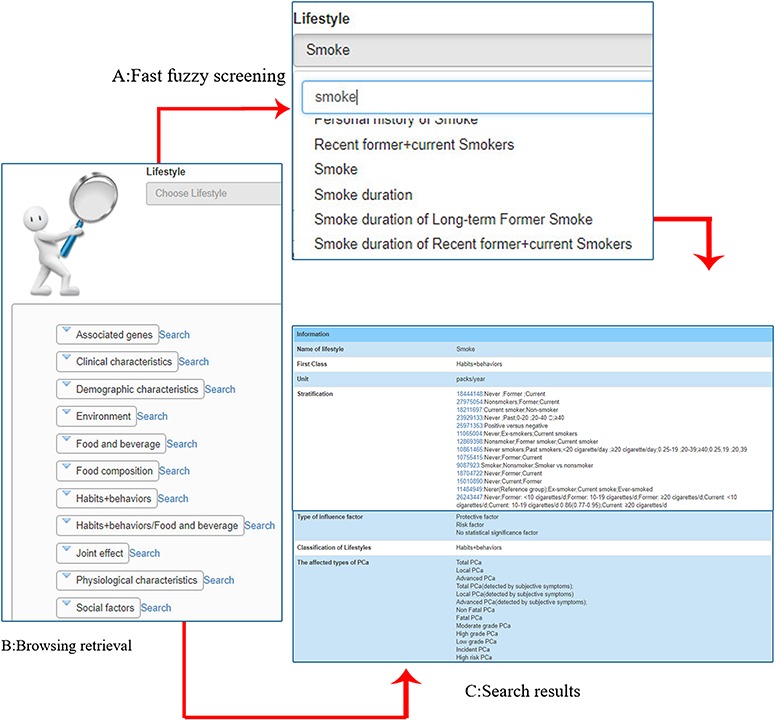
Demonstration of retrieval function. **A**: fast fuzzy screening (taking ‘smoke’ as an example); **B**: browsing retrieval; **C**: search results.

As one of the main functions of the database platform, the retrieval approach includes fast retrieval and browsing retrieval. ‘Fast fuzzy screening’ (Figure 4A) can intelligently match the words input by users and find the relevant lifestyle quickly. If the user’s goal is not clear, users can utilize the ‘Browsing retrieval’ function to browse preset list of categories, through step-by-step click to choose the lifestyle (Until the ‘Go to” button appears, click to see the corresponding search results) (Figure 4B). Information on each lifestyle in PCaLiStDB includes name, unit, classification, influence type, stratification, affected type of PCa and number of studies involved as well as the details (Figure 4C).

### Lifestyle-wide association studies of PCa

Looking at all the factors related to the occurrence of PCa in PCaLiStDB, we gained new insights into the occurrence of PCa at the level of lifestyle-wide association studies of PCa (PCa-LWAS).

**Figure 5:**
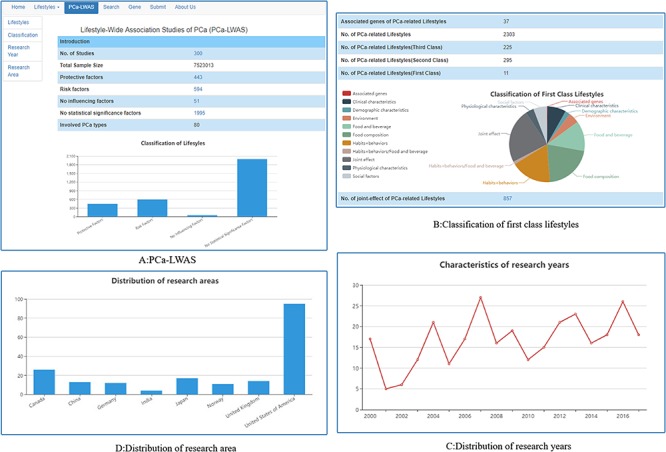
Lifestyle-wide association studies of PCa. (**A**) PCa-LWAS; (**B**) classification of first class lifestyles; (**C**) distribution of research years; (**D**) distribution of research areas.

The ‘PCa-LWAS’ menu lists the basic contents and related statistical results of PCaLiStDB. Users can browse directly or choose to view according to the left navigation area (Figure 5A). Three hundred studies covering 80 PCa types are included. The sample size reached up to 7523013. At present, the main research methods of lifestyle are randomized controlled trials and case-control studies. Most of them are based on questionnaires and few are intervention studies. Through the manual qualitative research, we screened 394 protective factors, 556 risk factors, 45 no influencing factors, 52 factors with contradictory views and 1977 unclear factors. At the same time, we applied the effect values and 95% CI to classify the impact degrees of the factors and marked with different number of symbol ‘☆’ to indicate their extent of correlation.

Thirty-seven PCa lifestyle-associated genes were screened out and standardized. In addition to the 2290 single-factor lifestyles, 856 joint-effects between two or more lifestyles were found; all these lifestyles have been classified into 11 first class (Figure 5B). From the distribution characteristics of research years, we can find that the total number of lifestyle research is rising year by year (Figure 5C). The study distribution was presented in Figure 5D, most of the studies is from USA, Canada and Japan. The well-known study cohorts are HPFS, EPIC, PROtEuS, ATBC, VITAL and NIH-AARP.

## Discussion

PCaLiStDB integrates the PCa-associated lifestyle with the standardized annotation. The database contains not only the routine lifestyles (22, 24) associated with the occurrence of PCa, such as diet, exercise and living habits but also the environment factors, disease phenotypes and drug information related to the lifestyles. Based on the collection, both contradictory associations and synergistic effects existed for the impact of lifestyle on PCa which indicates the lifestyle-disease associations are personalized and conditionally different (27). For example, the effect of dietary cadmium is related to waist circumference (28); the impact of height on PCa varies with family history, smoking and specific diseases (29). The roles of lifestyles certainly are affected by genetic situation, such as calcium intake is regulated by Vitamin D receptor (VDR) (15). Younger men who ever smoked (OR = 0.37, 95% CI: 0.20, 0.69) or carried the CYP3A43*3 variant (OR = 0.21, 95% CI:0.07, 0.63) has a significantly lower odds of developing PCa (30). Aspirin use is associated with lower PCa risk in male carriers of BRCA mutations (31). Vitamin E supplementation increased the risk for PCa in healthy men at a median of 7 years. It is worth noting that, undergoing 2*2 factorial trial design, the combination of vitamin E and selenium did not show their association with the occurrence of PCa (NNH = 105) (32).

Above observations indicate that the lifestyle-disease associations are personalized and heterogeneous. It is necessary to study the role of lifestyle in the context of macro-environment, even the genomics, so as to achieve systems biological level understanding of the lifestyle’s effect on PCa. The ultimate goal is to make the medical staff and patients get clear lifestyle guidance and achieve precision PCa prevention (20). It is hoped that users can acquire PCa-related lifestyle from PCaLiStDB and actively adopt the positive lifestyle for prevention and control of PCa.

### Advantages and disadvantages

Several strengths in this research should be highlighted. First of all, we systematically collected the up-to-date evidence for PCa-associated lifestyles. Second, through the PCa-LWAS, we can have comprehensive understanding of lifestyle stratification and sub-group information (33, 34). Third, to our knowledge, PCaLiStDB is the first lifestyle database established focusing on a specific disease. The annotation is standardized and the database is extensible for future updating and sharing.

Some issues need to be considered when applying and interpreting the PCaLiStDB. First, most of the studies are retrospective, but few are prospective interventional studies. Moreover, this reminds us that more interventional studies should be made to clarify the role of lifestyle. Second, we paid attention to and collected some lifestyle-related genes, such as MC1R (35, 36), BRCA (31) and VDR (37) but the in-depth mechanism researches are still needed. Finally, the role of these lifestyles in future disease prediction needs further precision model construction and validation studies.

Lifestyle medicine provides a feasible approach for the prevention and treatment of many modern chronic diseases (38). It plays a role between clinical medicine and public health, encouraging patients to adopt lifestyle modification for personalized healthcare (39, 40). Researches show that targeting lifestyle interventions may improve outcomes for chronic diseases, exert favorable effects on cancer treatment (41–43). In addition, the development of many kinds of eHealth/mHealth equipment and auxiliary methods will provide different ways for the conducting of lifestyle medicine.

## Conclusions and future directions

PCaLiStDB is the database focusing on PCa associated lifestyles. It integrates lifestyles and lifestyle-related factors of PCa and provides a comprehensive research platform for PCa-LWAS. It is devoted to accelerate the translation of LWAS discoveries into knowledge that improve the application of green clinical decision (lifestyle prescription) and self-intelligent healthcare (44). Through this platform, we expect to build precision predictive models for PCa prevention based on evidence-based personalized lifestyle data.

For the future maintenance of PCaLiStDB, we are developing prostate cancer lifestyle ontology (PCLiON) to standardize the description of prostate cancer associated lifestyles. Then we will analyze the joint effects of multi-lifestyle from PCaLiStDB and develop models for prediction of prostate cancer risk. PCaLiStDB will be updated annually and tools for the visualization of data will be added to the web database.

## Author contributions

The authors’ responsibilities were as follows—B.S. and Y.C. designed the research; X.L. constructed the database; Y.C., C.Y., Y.L. and L.Y. designed the pilot data extraction tables; Y.C., Y.Y., T.X., Z.Y. and Z.F. performed literature search, study selection and data extraction; Y.C. and X.L. conducted LWAS analyses and prepared the tables and figures; Y.C. and X.L. drafted the manuscript. B.S. supervised the study and revised the manuscript. All the authors completely consented with all the data in the study, critically revised the manuscript for important intellectual content and approved the final version.

## Data resource access

PCaLiStDB is available at: http://www.sysbio.org.cn/pcalistdb/
